# Rapid Evaluation of Vaccine Booster Effectiveness against SARS-CoV-2 Variants

**DOI:** 10.1128/spectrum.02257-22

**Published:** 2022-09-07

**Authors:** Hoi Lok Cheng, Sing Mei Lim, Huan Jia, Ming Wei Chen, Say Yong Ng, Xiaohong Gao, Jyoti Somani, Sharmila Sengupta, Dousabel M. Y. Tay, Patrina W. L. Chua, Abirami R., Sharon Y. H. Ling, Megan E. McBee, Barnaby E. Young, Hadley D. Sikes, Peter R. Preiser

**Affiliations:** a Singapore-MIT Alliance in Research and Technology, Singapore; b School of Biological Sciences, Nanyang Technological University, Singapore; c National University Hospital, Singapore, Singapore; d Department of Chemical Engineering, Massachusetts Institute of Technology, USA; e National Centre for Infectious Diseases, Singapore, Singapore; f Tan Tock Seng Hospital, Singapore, Singapore; g Lee Kong Chian School of Medicine, Nanyang Technological University, Singapore, Singapore; University Paris-Saclay, AP-HP Hópital Antoine Béclère, Service de Microbiologie, Institute for Integrative Biology of the Cell (I2BC), CEA, CNRS

**Keywords:** COVID, neutralizing antibodies, point-of-care test

## Abstract

As the COVID-19 pandemic continues, countries around the world are switching toward vaccinations and boosters to combat the pandemic. However, waning immunity against SARS-CoV-2 wild-type (WT) and variants have been widely reported. Booster vaccinations have shown to be able to increase immunological protection against new variants; however, the protection observed appears to decrease quickly over time suggesting a second booster shot may be appropriate. Moreover, heterogeneity and waning of the immune response at the individual level was observed suggesting a more personalized vaccination approach should be considered. To evaluate such a personalized strategy, it is important to have the ability to rapidly evaluate the level of neutralizing antibody (nAbs) response against variants at the individual level and ideally at a point of care setting. Here, we applied the recently developed cellulose pulled-down virus neutralization test (cpVNT) to rapidly assess individual nAb levels to WT and variants of concerns in response to booster vaccination. Our findings confirmed significant heterogeneity of nAb responses against a panel of SARS-CoV-2 variants, and indicated a strong increase in nAb response against variants of concern (VOCs) upon booster vaccination. For instance, the nAb response against current predominant omicron variant was observed with medians of 88.1% (*n* = 6, 95% CI = 73.2% to 96.2%) within 1-month postbooster and 70.7% (*n* = 22, 95% CI = 66.4% to 81.8%) 3 months postbooster. Our data show a point of care (POC) test focusing on nAb response levels against VOCs can guide decisions on the potential need for booster vaccinations at individual level. Importantly, it also suggests the current booster vaccines only give a transient protective response against some VOC and new more targeted formulations of a booster vaccine against specific VOC may need to be developed in the future.

**IMPORTANCE** Vaccination against SARS-CoV-2 induces protection through production of neutralization antibodies (nAb). The level of nAb is a major indicator of immunity against SARS-CoV-2 infection. We developed a rapid point-of-care test that can monitor the nAb level from a drop of finger stick blood. Here, we have implemented the test to monitor individual nAb level against wild-type and variants of SARS-CoV-2 at various time points of vaccination, including post-second-dose vaccination and postbooster vaccination. Huge diversity of nAb levels were observed among individuals as well as increment in nAb levels especially against Omicron variant after booster vaccination. This study evaluated the performance of this point-of-care test for personalized nAb response tracking. It verifies the potential of using a rapid nAb test to guide future vaccination regimens at both the individual and population level.

## INTRODUCTION

There is significant variation in the immune response to a particular vaccine between individuals. This can impact both the level, as well as duration, of a protective immune response (1–3). For this reason, individuals with a low antibody titers are recommended to consider a booster or a second course of vaccination in Singapore and Australia in cases of hepatitis B (4, 5). A personalized point of care (POC) immune testing strategy can represent an important tool in the current COVID-19 pandemic as it provides detailed information on the immune status of individuals and whether the need of additional booster vaccinations. Such an informed approach could also tackle vaccine hesitancy and broaden the global availability of vaccines.

Furthermore, the global spread of severe acute respiratory syndrome coronavirus 2 (SARS-CoV-2) has elicited variants with higher transmissibility and capability to evade immune responses, which have been referred to as variants of concern (VOCs). While the SARS-CoV-2 mRNA vaccines show excellent protection against the wild-type (WT) virus, there has been an increased risk for breakthrough and perhaps even severe infection from these VOCs recently, possibly due to waning and heterogeneous immunity in the population. In particular, the BNT162b2 vaccine effectiveness is reported to decline to 53% 4 months post-second-dose (P2) vaccination (6). The majority of vaccinated individuals in Singapore had received vaccination in early 2021, which puts them at > 6 months postvaccination and hence, considerably lower immunity against the virus (7). Recent BNT162b2 vaccine booster trials showed 95.6% efficacy among booster vaccinees who have completed their second dose 11 months earlier (8). In a bid to boost protection against VOCs, administration of vaccine booster to protect against the most recent VOCs (delta and omicron) is being implemented in countries like Israel, Germany, France, United States, and Singapore. According to the Morbidity and Mortality Weekly Report, the adjusted vaccine effectiveness of three-dose mRNA vaccine against emergency department and urgent care visits dropped from 87% to 31% at 5 months after the third dose (9). This situation led to the decision to give the fourth dose booster by the Israeli government (10). Nonetheless, a recent preprint has reported the fourth dose of BNT162b2 vaccine did not trigger a significantly better immune response compared with the third dose (11). This raised vaccine hesitation and questions toward the necessity of a fourth dose of any vaccine.

To precisely address the need of multiple booster doses, the risk of any new VOC, and the prioritization to high risked individuals, it is essential to have reliable information in relation to the variant-specific protective immune response at the individual level. Hence, we established a variant-specific rapid POC testing platform to evaluate the heterogeneity of any protective immune response, the waning effect postvaccination, and the potential immune escape of any VOC (12, 13). Using Singapore as an example, this testing platform will help guide the implementation of appropriate health care measures and resource allocation. Similar to the current hepatitis B vaccination, a personalized vaccine regimen against SARS-CoV-2 can be considered (14).

Neutralizing tests are a common approach to determine the protective immune response. SARS-CoV-2 neutralizing tests can assess the blocking activity of a sample against the binding of the viral spike protein to the human receptor. Only antibodies that can neutralize the binding, namely, neutralizing antibodies (nAbs), will be detected. As confirmed by Kongsuphol et al., only nAbs showed neutralizing activity in the cellulose pulled-down virus neutralization test (cpVNT) while non-neutralizing anti-receptor binding domain (RBD) IgG showed no response (12).

This study aims to evaluate the rapid POC use of the cpVNT as a tool for the evaluation of the nAb response thereby providing personalized information in relation to a decline in the nAb response after the second and third dose of a vaccine. In study 1, we measured the P2 waning of the two types of mRNA vaccines at different time points using cpVNT against WT. Study 2 evaluated nAb response against WT and VOCs after 1-month and > 3-months postbooster.

## RESULTS

### Study population.

**FIG 1:**
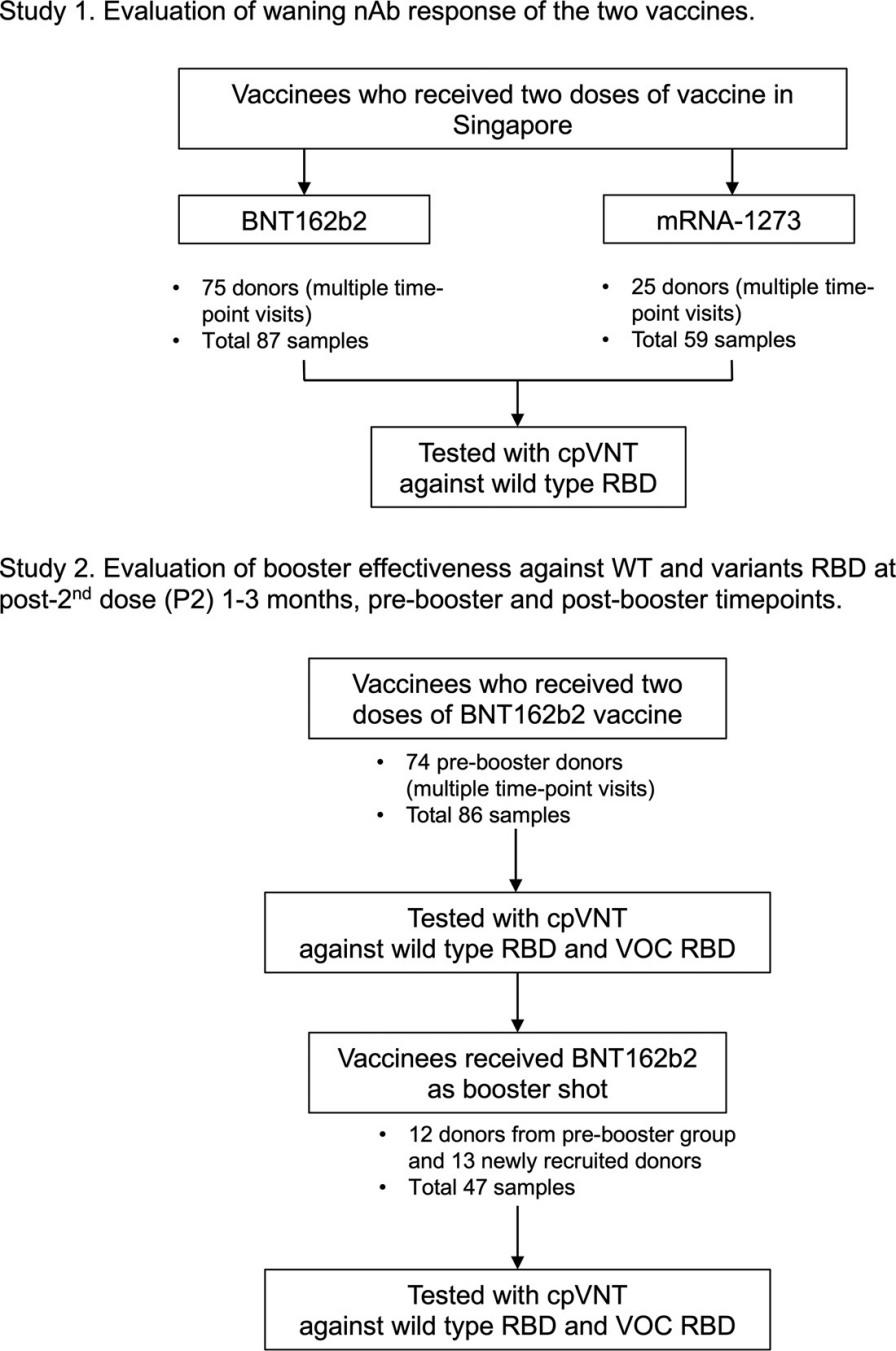
Selection of participants and testing. This study involved persons who were fully vaccinated with two doses of BNT162b2 or mRNA-1273, and persons who received one dose of BNT162b2 as booster after being vaccinated with two doses of BNT162b2 before April 5, 2022.

For the waning evaluation in study 1, we collected a total of 146 whole-blood samples from 100 volunteers (multiple visits) with 87 samples having received two doses of the BNT162b2 and 59 received the mRNA-1273 vaccine. There was a total of 86 samples from 74 of the BNT162b2 vaccinees that fell into the P2 1 to 3 months and prebooster subgroups in study 2. Twenty-five volunteers, including 13 newly recruited participants, contributed to 47 BNT162b2 postbooster samples for the booster effectiveness evaluation in study 2. Forty-five plasma samples were separated from study 2 samples and used for plasma-based cpVNT tests against the VOCs, including mu and omicron that is presented in Fig. S3. The study participation is shown in Fig. 1.

### Post-second-dose nAb waning of BNT162b2 and mRNA-1273 vaccinees.

**FIG 2:**
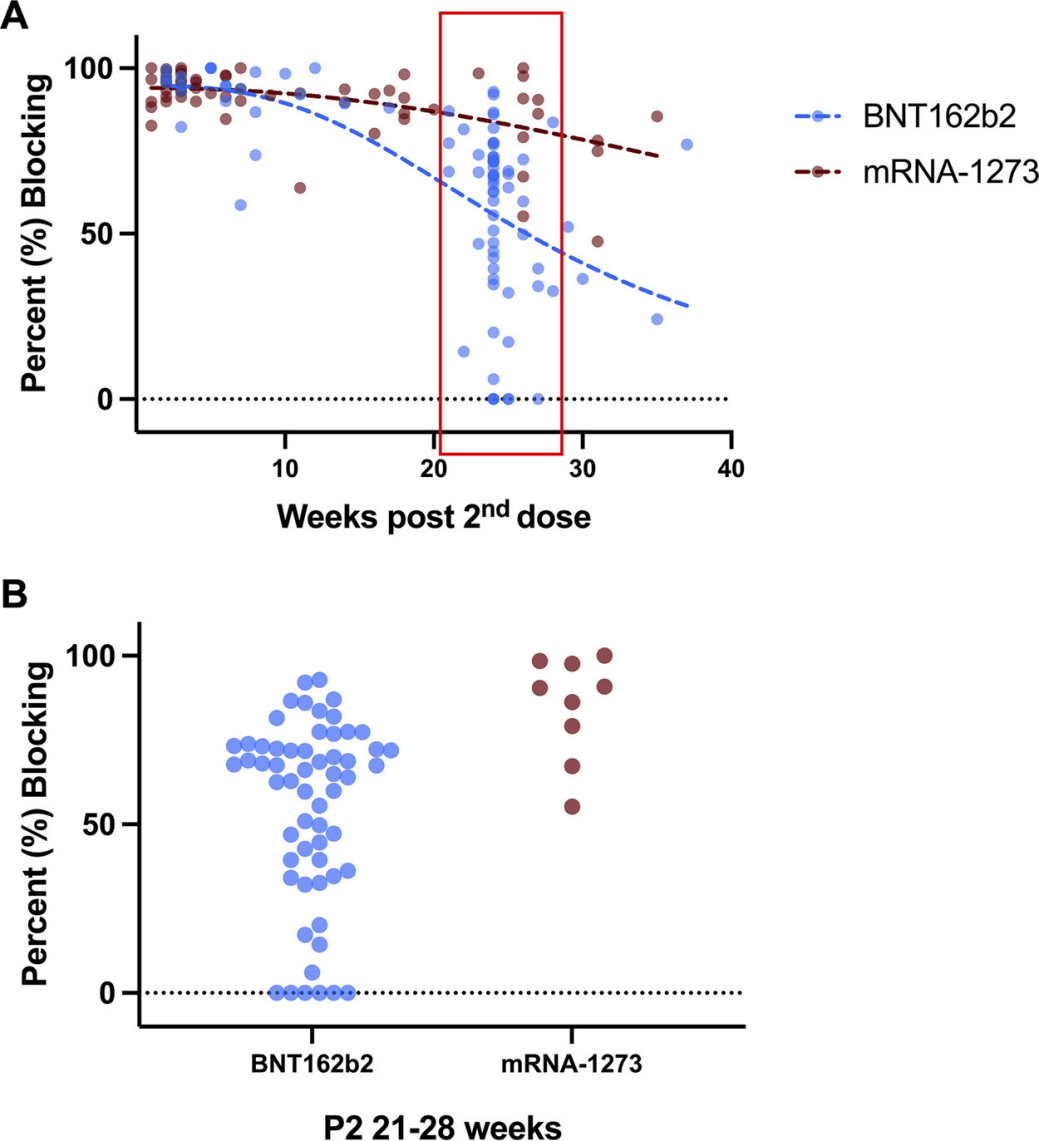
Prebooster waning trend of the mRNA vaccines and the diverse nAb response of individuals. (A) The % nAb blocking from vaccinated whole-blood samples stratified by vaccine type and weeks post-second-dose were measured against WT. A total of 85 samples completed two doses of BNT162b2 vaccine while 59 samples were previously vaccinated by mRNA-1273. Each dot represents the average % blocking of one sample. A least squares nonlinear regression model with no weighting was used to generate the waning trend as dotted lines. Extra sum-of-squares F test was performed to calculate the significance of difference between the two trendlines and the *P* value is given in the main text. Samples from P2 21 to 28 weeks (in red box) are selected for individual nAb response analysis; (B) The % nAb blocking of samples from P2 21 to 28 weeks were grouped by vaccine type and shown on scatterplot. Selected samples consist of 57 BNT162b2 samples and nine mRNA-1273 samples.

The P2 waning trends of the two vaccine types were assessed by plotting the average percent nAb blocking of each sample against weeks P2 vaccination (Fig. 2A). Least squares nonlinear regression model was used to generate the waning trend line for both vaccines. A significant difference with an F ratio of 12.1 (*P* < 0.0001) was found between the two waning trend lines. Currently, a booster shot is recommended around 6 months P2 for increased immunity by the Singapore national booster vaccination program. Thus, samples from P2 21 to 28 weeks were isolated and grouped by vaccine type for the individual nAb response analysis (Fig. 2B). At this time point, there is a large variation in individuals’ nAb response with the percent blocking of BNT162b2 samples (*n* = 57) ranging from 0.0% to 92.8%, while the range of mRNA-1273 samples (*n* = 9) was 55.3% to 100.0%. This heterogeneity in the longevity of the nAb response was also observed in stored plasma samples from the same individuals (Fig. S3).

### Booster effectiveness against SARS-CoV-2 WT, delta, beta, gamma, and omicron variants.

**FIG 3:**
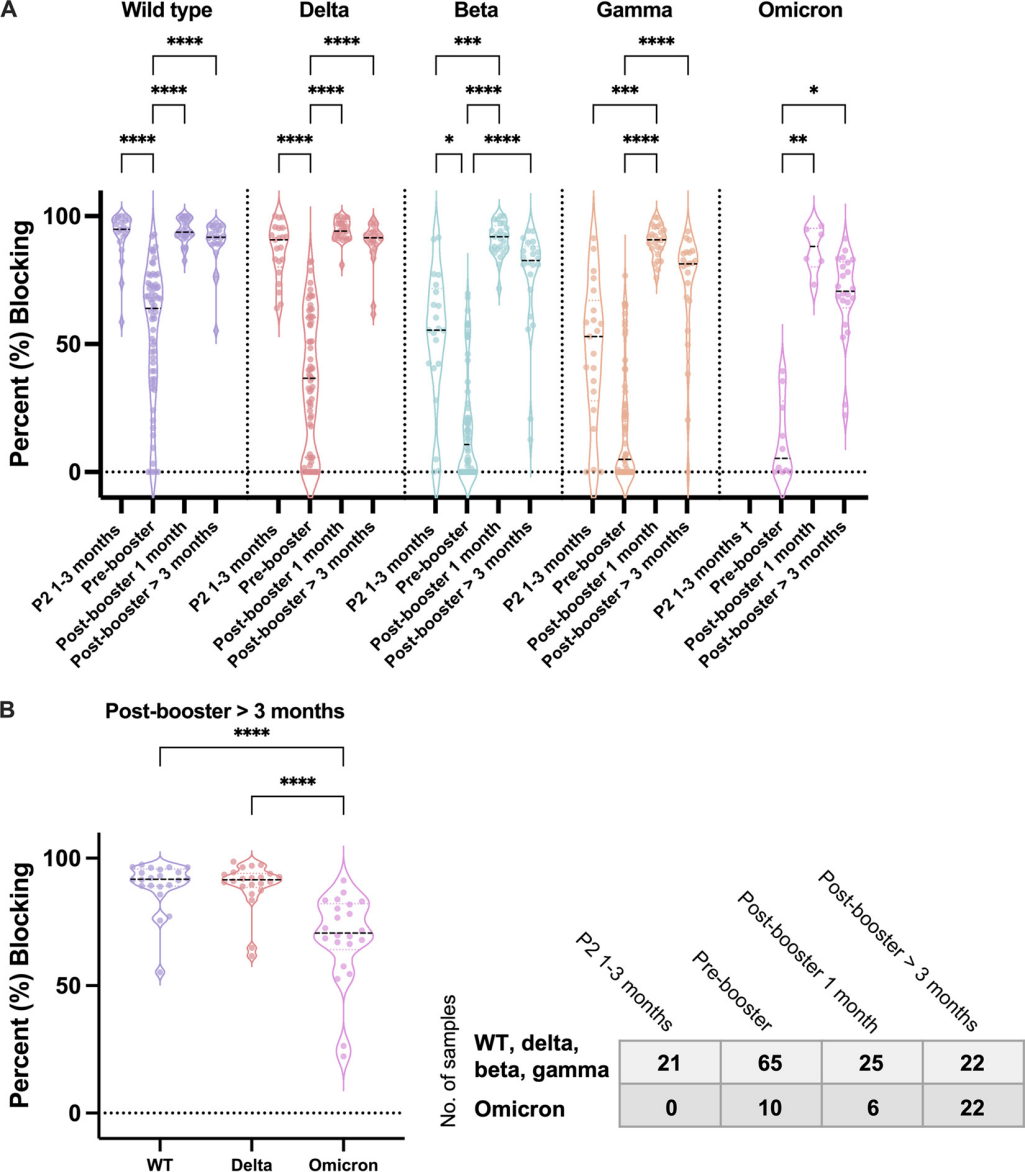
Booster effectiveness measured by whole-blood-based cpVNT against SARS-CoV-2 WT, delta, beta, gamma, and omicron variants. (A) The nAb response represented as % blocking was measured against WT, delta, beta, gamma, and omicron RBD from samples at post-second-dose (P2) 1 to 3 months, prebooster which is P2 5 months onwards, postbooster 1 month, and postbooster > 3 months with whole-blood-based cpVNT. (B) A comparison of nAb levels of the 14 postbooster > 3 months samples against WT, delta, and omicron RBD. Each dot represents the average % blocking of one sample. The medians are indicated as dotted lines. The table represents number of samples tested from each time point. Kruskal-Wallis tests between different time points in (A), and Friedman test between WT, delta, and omicron in (B), with Dunn’s multiple comparisons tests were performed, where *, *P* < 0.05; **, *P* < 0.01; ***, *P* < 0.001; ****, *P* < 0.0001. †No P2 1 to 3 months whole-blood sample was available for test against omicron.

We compared the nAb response against WT and the VOCs (delta, beta, gamma, and omicron variants) among P2 1 to 3 months, P2 prebooster, postbooster 1 month, and postbooster > 3 months subgroups of BNT162b2 vaccinees. We observed the median percent blocking against WT and VOCs at P2 1 to 3 months varied considerably with nAb against WT and delta, achieving higher levels compared with beta or gamma (Fig. 3A). Moreover, there was a significant drop in the median percent blocking against WT and VOCs at the prebooster time point compared with the P2 1 to 3 months (Fig. 3A). Following a booster dose of BNT162b2, the median percent blocking showed a significant increase to over 90% against WT as well as the VOCs, including omicron. Compared with P2 prebooster, the increases in percent nAb blocking after booster are 29.8% for WT (63.9→93.7%, *P* < 0.0001), 57.4% for delta (36.7→94.1%, *P* < 0.0001), 81.3% for beta (10.7→92.0%, *P* < 0.0001), 85.9% for gamma (4.9→90.9%, *P* < 0.0001), and 82.8% for omicron (5.4→88.1%, *P* = 0.0032). Comparison of P2 1 to 3 months and postbooster response against the beta and gamma variants showed an increased nAb response against these two variants with the differences between P2 1 to 3 months and postbooster being 36.5% (55.5→92.0%, *P* = 0.0003) for beta and 37.9% (52.9→90.8%, *P* = 0.0001) for gamma. This is in concordance with the Pfizer study using a virus neutralization test and shows that the booster can achieve higher levels of nAb response against VOCs than that achieved by the initial two-dose vaccination (15). Importantly, there is significant heterogeneity in the prebooster nAb response between individuals against VOCs (Fig. 3A) similar to that observed in the waning study against WT (Fig. 2B). The range of percent blocking at prebooster period was from 0.0% to 82.5% against delta, from 0.0% to 69.8% against beta, from 0.0% to 76.7% against gamma, and from 0.0% to 39.4% against omicron. Comparable changes in the levels of nAbs against WT and VOCs were also observed when using stored plasma samples (Fig. S3), once again providing overall confidence in the POC approach utilized here.

Evaluation of the persistence of nAbs showed that 3 months after the booster dose, the nAb response against WT and delta were still high with medians of 91.8% (95% CI = 89.1% to 95.6%) and 91.5% (95% CI = 88.9% to 93.9%) while the medians against beta and gamma had dropped to 82.7% (95% CI = 71.3% to 87.6%) and 81.3% (95% CI = 67% to 85.8%), respectively. A lower median nAb response of 70.7% (95% CI = 66.4% to 81.8%) was observed against the current predominant omicron variant (Fig. 3A). Comparison of the persistence of the nAb levels against the currently dominant VOCs showed there is a noteworthy drop in the median percentage blocking against omicron compared with either WT (91.8% to 70.7%, *P* < 0.0001) or delta (91.6% to 70.7%, *P* < 0.0001) postbooster > 3 months, suggesting a long lasting nAb response against more immunologically diverse VOCs may be difficult to achieve while boosting with the current mRNA vaccines that are based on the WT virus (Fig. 3B).

## DISCUSSION

As the world transitions back to a postpandemic normal, knowledge on immune status at both the population and individual level becomes paramount to control COVID-19 resurgence. Antibody-mediated humoral immunity and T cell-mediated cellular immunity work together to protect an individual from infection and morbidity. The presence of high levels of nAbs has been shown to correlate well with prevention of symptomatic infections and is predictive of protection against the disease (16–18). Additionally, immunodeficient patients may rely heavily on detectable levels of circulating nAb as an indicator of protection level from disease. Assessing the nAb response toward VOCs is helpful in determining whether new strains of virus can escape immunity in the community, regardless of their vaccination status. Previously, we established a blood-matrix-compatible point-of-care test, cpVNT, that correlates well with plasma-based ELISA and pseudovirus neutralization test (pVNT) results and provides a reliable correlate between plasma-based and whole-blood-based nAb response (12, 13). The cpVNT, as well as the other neutralization tests, detects nAbs using a competitive assay format. These tests measure neutralizing activity from samples and do not differentiate nAbs induced by vaccination versus infection. Compared with the most recognized lab-based pVNT, our cpVNT showed a sensitivity of 100% (95% CI = 47.8% to 99.9%) and a specificity of 66.7% (95% CI = 38.4% to 88.2%). When we compared the cpVNT with the commercially available sVNT Genscript cPass, the sensitivity was 81.5% (95% CI = 61.9% to 93.7%) and the specificity was 100% (95% CI = 81.5% to 100%) (13). One of its key features is that the cpVNT can evaluate the competition between nAb and angiotensin converting enzyme-2 (ACE-2) against the RBD with a turnaround time under 10 min outside of a laboratory setting. As demonstrated in this study, this rapid test is readily extended for population-level nAb response monitoring against the RBD of any VOC by replacing the WT RBD used in the neutralizing assay with a variant’s RBD.

Here, we report the evaluation of vaccine and booster immunization protocols in developing and maintaining nAbs not only against WT but also VOCs. The cpVNT results indicate the initial generation of high levels of nAbs against WT RBD after a two-dose vaccination regimen with either BNT162b2 or the mRNA-1273 vaccine. However, the levels of nAbs decline to approximately 50% after 5 to 6 months for BNT162b2 while remaining above 70% for mRNA-1273 at 35 weeks P2 vaccination. The observation of BNT162b2 samples is in line with other reports, which measure overall immunity, IgG, and neutralizing antibody (pVNT) (7, 19–21). This result also agrees with previous mRNA-1273 reports (22–24). A striking finding in our study is the diverse nAb response generated as well as differences in waning over time that is observed between individuals (Fig. 2; Fig. S2). The clear differences between individuals suggest a personalized booster vaccination schedule, which can be easily assessed using the cpVNT as a reliable alternative to lab-based neutralization tests, could be considered to guide public health policy.

The data also show that two doses of BNT162b2 induce high levels of nAbs against the WT as well as delta variant with lower levels of nAbs being observed against beta, gamma, and omicron. As observed for WT, the levels of nAbs against VOCs significantly decline over time and in many individuals are close to 0% inhibition before booster vaccinations. The reduced level of nAbs prebooster are particularly striking for beta, gamma, and omicron suggesting protection against these variants is potentially compromised. This is consistent with the study by Cele et al. and Wilhelm et al. who reported a significant drop of neutralizing titer against the omicron variant compared with D614G and delta, respectively (25, 26). Booster vaccination with BNT162b2 leads to a significant increase in nAb responses against the tested panel of VOCs, including omicron, achieving medians close to 90%. Our data are somewhat in contrast to the study by Wilhelm et al. showing that while postbooster sera samples showed a higher median titer than the P2 samples, only 58% of the BNT162b2 booster vaccinees showed neutralization response against omicron half a month after the vaccination. However, more rapid decrease in nAb level was observed against omicron compared with WT and delta 3 months after the booster vaccination (Fig. 3B). The reduced nAb response against omicron variant was further confirmed by comparing the nAb response against WT or delta of the same individual samples from various vaccination time points, as shown in Fig. S4. The results together raise public health concerns regarding WT-based vaccines.

Correlation between the nAb response and the protection against infection as well as severe symptoms has been suggested by various published data, which indicates low nAb response may increase risk of infection by new VOCs. In this study, we utilized the rapid POC cpVNT to measure and analyze the P2 waning and booster dose effectiveness of the mRNA vaccines in Singapore. Our data supports the current Singapore implementation of the booster vaccination program, especially in consideration of the prevalence of VOCs among the global reported infections (27). Additionally, the cpVNT measurement of nAbs blocking SARS-CoV-2 RBD–ACE2 interactions distinguishes individual nAb responses to specific RBDs. Aligning with other reports, our data indicate the WT-based vaccine booster dose can restore nAbs levels against the WT and partial responses to the current variants. However, long-term protection against the predominant VOC remains a problem. Huge heterogeneity of nAb levels among the individuals poses an extra challenge. Our cpVNT results indicate that a more personalized, rather than a population data-based vaccination strategy, could be beneficial to more effectively manage the limited vaccine resources globally and to also more effectively address vaccine hesitancy especially in relation to multiple booster shots (28–30). With a POC test that is as quantitative as the common lab-based assays, the testing strategy used here demonstrates its utility as a triage for future vaccine administration need. Long-term evaluation of nAb response postvaccination, heterologous or homologous boosting phase against emerging VOCs can provide a powerful tool to guide public health policies against SARS-CoV-2.

In this study, we have demonstrated the cpVNT platform is practical for clinical usage and ready for adoption. Importantly, it can be easily adapted for any new threatening VOCs’ RBD to evaluate the risk of immune escape. Going forward, the technology can be developed into a multiplex testing format to evaluate neutralization against multiple trending variants with a single drop of finger stick blood sample. At the same time, the underlying approach to the cpVNT platform can be utilized to evaluate nAb against other viral infectious diseases of concern in the future.

## MATERIALS AND METHODS

### Participants and sample collection.

This study recruited volunteers who were healthy adults ages 21 to 75 and had completed the vaccination regimen with BNT162b2 vaccine or mRNA-1273 vaccine as well as those who received their booster shots. National Centre of Infectious Disease (NCID) provided whole-blood samples from healthy volunteers who had received two doses of BNT162b2 vaccine 3 to 6 months prior. Finger stick blood samples were collected using Haim Winnoz blood collection device while venous blood samples were collected by phlebotomists. There was no report of any comorbidities or breakthrough infection from these volunteers during the study period. For study 1, a total of 100 volunteers were recruited and contributed one or more samples at different visits, which fall into the two groups of different vaccine type and the subgroups stratified by weeks postvaccination (Fig. 1). For study 2, a total of 87 volunteers were recruited and contributed one or more samples at different visits, classified according to the three subgroups of P2 1 to 3 months, prebooster (P2 5 months onwards), postbooster within 1 month, and postbooster > 3 months (Fig. 1). Plasma samples were separated from the whole-blood samples of sufficient volume by centrifugation and stored in −80°C for any further testing.

### Vaccination schedule.

As of November 30, 2021, 86% of the total population in Singapore had completed the full regimen of two doses of COVID-19 vaccinations (31). Since September 14, 2021, those who received the two primary doses at least 6 months prior became eligible to receive either the Pfizer-BioNTech/Comirnaty (BNT162b2) vaccine or the Moderna/SPIKEVAX (mRNA-1273) vaccine as booster.

### Study design.

The prospective cohort study was designed to evaluate the SARS-CoV-2 vaccines’ waning until booster and booster effectiveness at increasing nAb levels and to VOC over 6 months. The neutralizing antibody response data generated were sorted based on vaccine types and number of doses. Samples with two doses of mRNA-1273 vaccine were planned for nAb response against WT RBD. Due to the earlier deployment of the BNT162b2 vaccine, only BNT162b2 vaccinees were selected for booster effectiveness study. P2 and postbooster samples from BNT162b2 vaccinees were planned for nAb response against WT, delta, beta, and gamma variant RBD. Time points of weeks postvaccination were used as subgroups for waning observation. For booster effectiveness, the time points of P2 1 to 3 months, 5 months onwards (prebooster), postbooster 1 month, and postbooster > 3 months were used for better analysis of waning nAb response before and after administration of a booster dose. No P2 1 to 3 months and fewer whole-blood samples were available for the cpVNT test against omicron because of the late emergence of this variant. Part of the data from P2 1 to 3 months samples have been reported in our previous publication (13). Booster effectiveness results that were stratified by demographic groups of gender and age are shown in Fig. S1A to D. No significant difference was observed between these demographic groups after booster vaccination and thereby in the main study, all demographic groups were combined to have greater sample sizes. The characteristics are summarized in Table S1.

Whole-blood or plasma samples were subjected to duplicate tests with our modified cpVNT against WT, delta, beta, gamma, mu, and omicron variant RBD (12, 13). The working principle and assay protocol of the cpVNT can be found in the online supplementary material. The neutralizing antibody response was measured as percent (%) blocking that inhibited the fluorescence signal generation and was calculated according to our established equation 1:

(1) $$\%\;\mathrm{Blocking}=\left(1-\frac{\mathrm{Voltage}\;\mathrm{output}\;\mathrm{of}\;\mathrm{test}\;\mathrm{spot}-\mathrm{Baseline}}{\mathrm{Voltage}\;\mathrm{output}\;\mathrm{of}\;\mathrm{Negative}\;\mathrm{Cont}\text{r}\mathrm{ol}\;\left(\mathrm{NC}\right)-\mathrm{Baseline}}\right)\times \;100\text{\%}$$

The values of baseline and negative control (NC) were established and described in our previous reports (13). Data of the cpVNT against WT from both P2 cohorts of BNT162b2 and mRNA-1273 was used for waning analysis. Data of the cpVNT against WT, delta, beta, gamma, and omicron variants from P2 1 to 3 months, prebooster, postbooster 1 month, and postbooster > 3 months subgroups of BNT162b2 vaccine was analyzed for assessing the booster effectiveness.

### Statistics.

For the primary analysis of nAb waning response regarding the two vaccine types, we estimated a reasonable sample size over 50 samples of each vaccine type based on the rules of thumb to generate sigmoidal waning model using least squares nonlinear regression with no weighting (32). Extra sum-of-squares F test was performed for the null hypothesis that the combined data set can be represented by one regression line.

For the secondary analysis of booster effectiveness regarding persons who received three doses of BNT162b2, a hypothesis of percent blocking 1 month post-third-dose (postbooster 1 month) being boosted to medians of 95%, 90%, and 90% against delta, beta, and gamma variants was made. We anticipated a median percent blocking of 35% against the delta variant at prebooster, and medians percent blocking of 55% and 50% against beta and gamma variants at P2 1 to 3 months. We hypothesized a significant boost of protection against delta variant compared with the prebooster period, and a significant difference between the second and the third dose of BNT162b2 vaccine against beta and gamma variants. Minimal total sample sizes to reject the null hypothesis of no difference between these subgroups were 12, 26, and 21, respectively, with a power of 80% and a two-sided error rate of 0.05.

The estimations were based on our previous report (13). All sample collections were planned to meet the prespecified criteria. The sample size calculations were based on the reported method regarding difference in medians for positively skewed outcomes (33).

The average of the duplicate test of a sample contributed to one data point in the specific subgroup based on time point and vaccine type. For the primary analysis of waning, we performed comparisons of the two regression trends of BNT162b2 and mRNA-1273 vaccines using extra sum-of-squares F test. For the secondary analysis of booster effectiveness, subgroup medians were calculated and the subgroups of different time points against a particular variant were compared using Kruskal-Wallis tests with Dunn’s multiple comparisons tests. Comparison of postbooster > 3 months samples against WT, delta, and omicron was analyzed using Friedman test with Dunn’s multiple-comparison test. All statistical analysis was carried out using GraphPad Prism version 9.1.1.

### Study approval.

This study was subjected to relevant ethical regulations and approval from the Institutional Review Board of Nanyang Technological University (IRB-2021-04-020). The whole-blood samples provided by National Centre of Infectious Disease (NCID) were approved under DSRB 2012/00917. All participants provided informed consent prior to sample collection.
