# Supplementary material for: Rapid Evaluation of Vaccine Booster Effectiveness against SARS-CoV-2 Variants
Source: Microbiol Spectr. 2022 Sep 7;10(5):e02257-22. doi: 10.1128/spectrum.02257-22 (PMC9602425; doi:10.1128/spectrum.02257-22)
Supplement: Supplemental file 1 — Supplemental methods, Table S1 and Fig. S1 to S4. Download spectrum.02257-22-s0001.pdf, PDF file, 3.6 MB [file spectrum.02257-22-s0001.pdf]

Rapid evaluation of vaccine booster effectiveness against SARS-CoV-2 variants

Hoi Lok Cheng,[1] Sing Mei Lim,[1] Huan Jia,[1] Ming Wei Chen,[2] Say Yong Ng,[1] Xiaohong Gao,[2] Jyoti Somani,[3] Sharmila Sengupta,[3] Dousabel M.Y. Tay,[4] Patrina W.L. Chua,[1] R Abirami,[1] Sharon Y.H. Ling,[1] Megan E. McBee,[1] Barnaby E. Young,[5,6,7] Hadley D. Sikes,[1,4] and Peter R. Preiser[2]

[1]Singapore-MIT Alliance in Research and Technology, Singapore. [2]School of Biological Sciences of Nanyang Technological University, Singapore. [3]National University Hospital, Singapore. [4]Department of Chemical Engineering of Massachusetts Institute of Technology, US. [5]National Centre for Infectious Diseases, Singapore. [6]Tan Tock Seng Hospital, Singapore. [7]Lee Kong Chian School of Medicine of Nanyang Technological University, Singapore.

Authorship notes: HLC, SML, and HJ are co-first authors.

HDS and PRP are co-corresponding authors. Emails: sikes@mit.edu; prpreiser@ntu.edu.sg.

Conflict of interest: HJ, MEM, HDS, and PRP are the cofounders of Thrixen Pte Ltd which focuses on development of the neutralization assay technology presented in this study.

Supplementary Information

Methods

Protein production and purification

The procedures of expression and purification of soluble extracellular fragment of human ACE2 (residues 19–615), wildtype, delta, beta, and gamma SARS-CoV-2 Spike RBD fused to cellulose binding domain were carried out as described in Kongsuphol et al. and Lim et al..[1,2] For the new SARS-CoV-2 variants' RBD-CBD, mu (p.R346K E484K N501Y) and omicron (p.G339D S371L S373P S375F K417N N440K G446S S477N T478K E484A Q493R G496S Q498R N501Y Y505H) were expressed in Expi293F cells (Thermo Fisher Scientific, A1435101) according to the supplier's protocol. The same protein purification protocol was used, including affinity chromatography with Ni-NTA cartridges (Qiagen, 1046323) and size exclusion chromatography with HiLoad 16/60 Sephadex 200 (Cytiva) in 20 mM HEPES pH 7.5, 300 mM NaCl, 10% glycerol. The His-MBP tag of RBD-CBD was removed by incubation with TEV protease overnight in 1:40 mass ratio at 4°C, followed by purification using reverse affinity chromatography with HisPur-Ni-NTA resin in 20 mM HEPES pH 7.5, 300 mM NaCl, 10 mM Imidazole. Purified RBD-CBD products were concentrated and stored in 20 HEPES pH 7.5, 300 mM NaCl, 10% glycerol and 0.5 mM TCEP at -80°C freezer before use.

Conjugation of monoFc-ACE2 with Alexa Fluor 594

The Alexa Fluor® 594 conjugated monoFc-ACE2 (ACE2-AF594) was prepared using the Alexa Fluor® 594 Conjugation Kit (Fast) - Lightning-Link® (abcam, ab269822). To perform one conjugation reaction, 10 µL of the modifier reagent was added to 100 µL of 1 mg/mL of monoFc-ACE2 in Phosphate Buffer Saline (PBS) pH 7.6. The 110 µL of mixture was then transferred to the Alexa Fluor® 594 Conjugation Mix, mixed well and incubated at room temperature for 30 minutes in the dark. Reaction was stopped by an addition of 10 µL of the quencher reagent with 15 minutes incubation in the dark. The conjugated product was stored in aliquots of 5 µL at – 80°C freezer before use.

Assay protocol of cellulose pulldown virus neutralization test (cpVNT)

The cpVNT testing cassette was assembled with 1 layer of Whatman No. 1 chromatography paper (GE healthcare, #3001-861) as a cellulose test strip, and 2 layers of Whatman gel blotting paper, Grade GB005 (GE healthcare, #10426981) as absorbent material in the

cassette housing (Racer Technology Pte. Ltd.). The test spot and reagent control spot were blocked with 5 µL of 5% Bovine Serum Albumin (BSA) in PBS pH 7.6 and the control spot was subsequently treated with 5 µL of 5 µM RBD-CBD in PBS pH 7.6, 1% BSA. To perform one test, 20 µL of whole blood sample was mixed with 20 µL of 10 nM RBD-CBD in PBS pH 7.6, 1% BSA and incubated for 3 minutes. For each VOC that was tested, the RBD-CBD protein used is the respective VOC RBD-CBD. 40 µL of 5 nM Alexa Fluor594 conjugated monoFc-ACE2 (ACE2-AF594) in PBS pH 7.6, 1% BSA was then added to the mixture and incubated for another 5 minutes. The reaction mixture was applied to the test spot and the control spot with 40 µL each. After the mixture was completely absorbed, both spots were washed with 40 µL of PBS pH 7.6. Finally, the cassette was transferred to the Atto Testbed, a customized reading unit described in Lim et al., for fluorescence measurement.[2] The entire assay procedure was performed at room temperature.

S1 Table. Demographic Characteristics of the Recruited Volunteers.

Characteristics	BNT162b2 cohort				mRNA-1273 cohort		
	1-3 months (N=18)	4-6 months (N=47)	> 6 months (N=19)	BNT162b2 as booster (N=25)	1-3 months (N=18)	4-6 months (N=11)	> 6 months (N=12)
Age category							
21-45 yr	13	31	9	6	15	11	6
46-75 yr	5	16	10	19	2	0	6
Unknown	0	0	0	0	1	0	0
Sex							
Female	6	29	11	12	9	5	8
Male	12	18	8	13	9	6	4

S1 Figure. Demographic booster effectiveness against SARS-CoV-2 WT, delta, beta, gamma, and omicron variants on whole blood-based cpVNT.

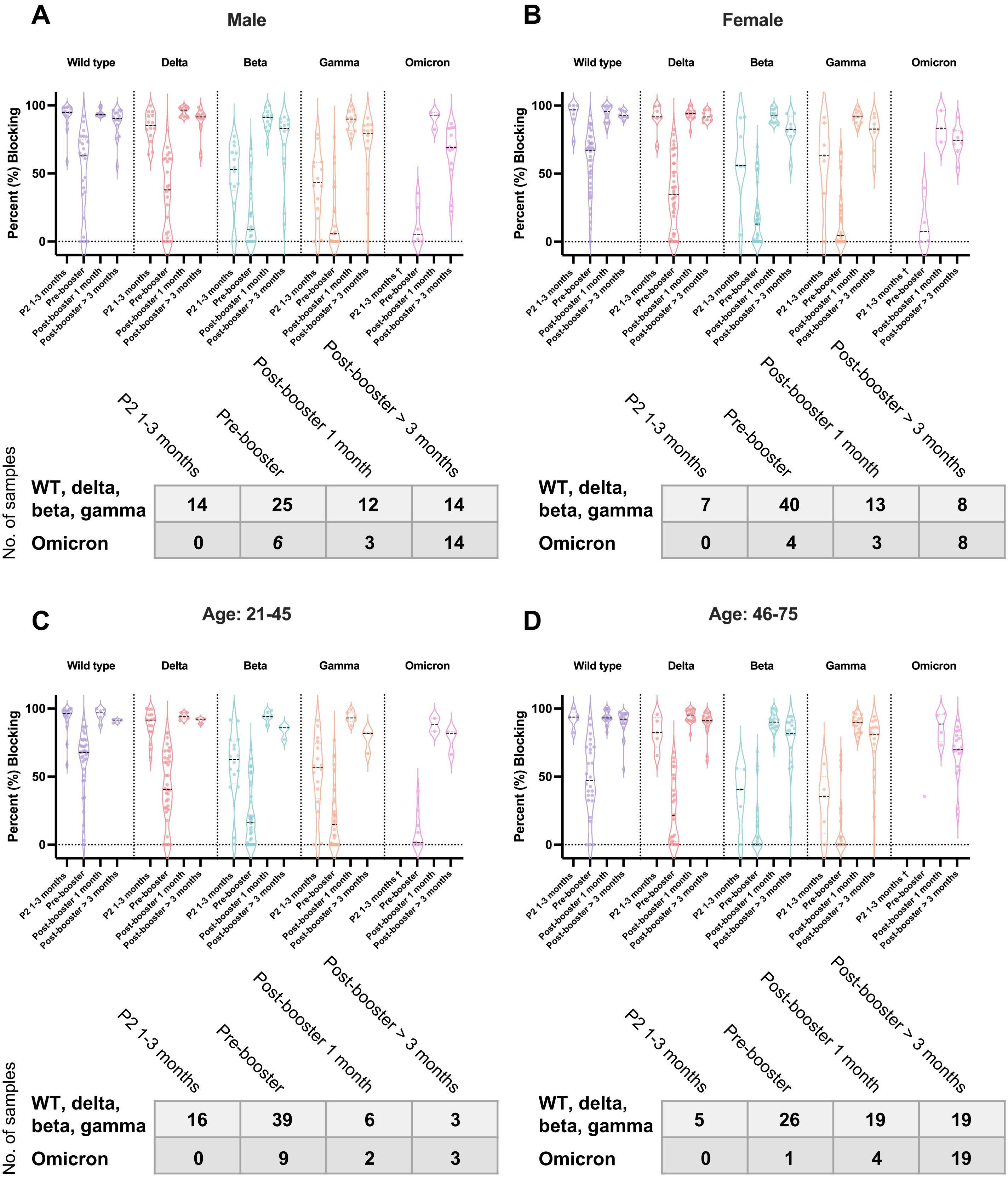

(A) Male

No. of samples	P2 1-3 months	Pre-booster	Post-booster 1 month	Post-booster > 3 months
WT, delta, beta, gamma	14	25	12	14
Omicron	0	*6*	3	14

(B) Female

No. of samples	P2 1-3 months	Pre-booster	Post-booster 1 month	Post-booster > 3 months
WT, delta, beta, gamma	7	40	13	8
Omicron	0	4	3	8

(C) Age: 21-45

No. of samples	P2 1-3 months	Pre-booster	Post-booster 1 month	Post-booster > 3 months
WT, delta, beta, gamma	16	39	6	3
Omicron	0	9	2	3

(D) Age: 46-75

No. of samples	P2 1-3 months	Pre-booster	Post-booster 1 month	Post-booster > 3 months
WT, delta, beta, gamma	5	26	19	19
Omicron	0	1	4	19

The nAb response response data against WT, delta, beta, gamma, and omicron variant from Figure 3 were stratified by demographic factors into groups of (A) Male, (B) Female, (C) Age: 21-45, and (D) Age: 46-75.

S2 Figure. Individual tracking of waning nAb response after 2-doses vaccination using cpVNT as a POC assessment.

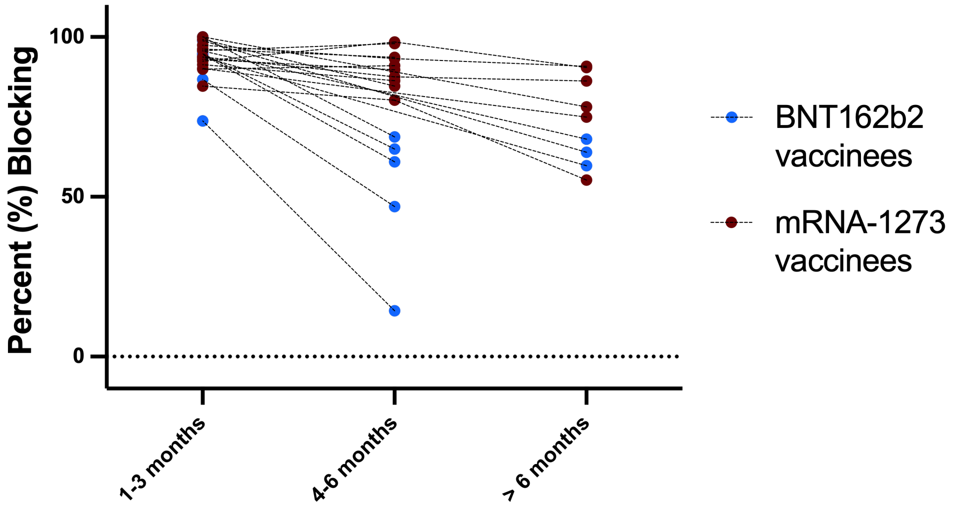

Individual nAb response assessment using whole blood-based cpVNT at there timepoints including 1-3 months, 4-6 months, and > 6 months post 2nd dose vaccination. There were 8 BNT162b2 vaccinees (blue dots) and 12 mRNA-1273 vaccinees (brown dots) contributed whole blood samples at multiple timepoints post-2nd dose. The dotted lines connect the multiple visits from the same individual.

S3 Figure. Booster effectiveness measured by plasma-based cpVNT against SARS-CoV-2 WT, delta, mu, and omicron variants.

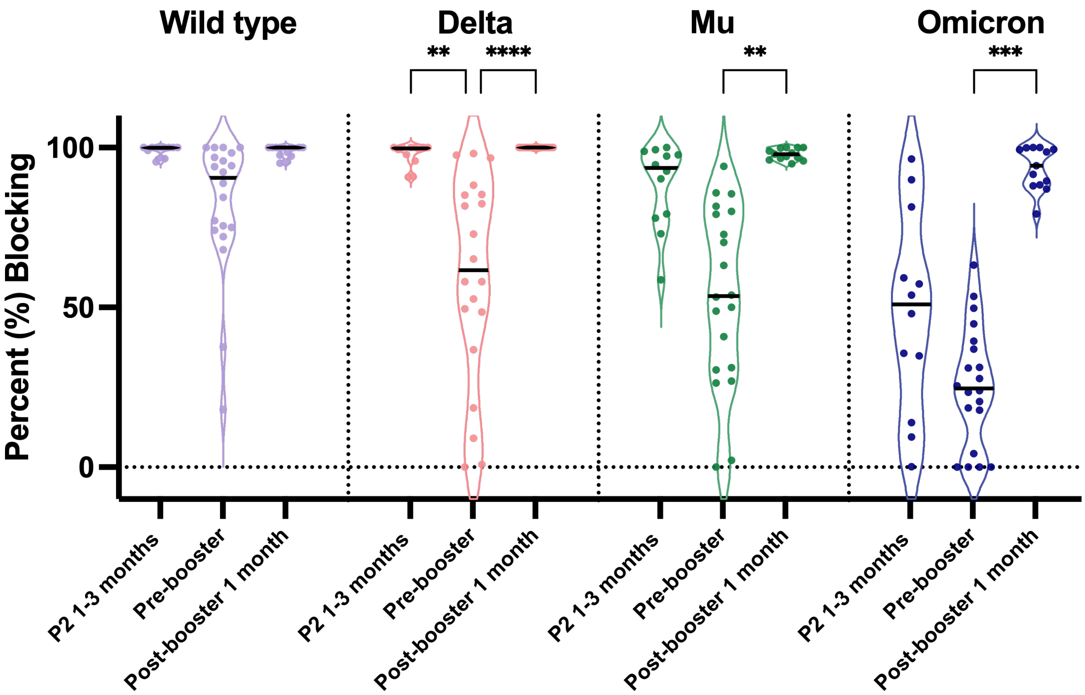

No. of samples

	P2 1-3 months	Pre-booster	Post-booster 1 month
**Plasma**	**12**	**20**	**13**

The nAb response represented as % blocking was measured against wild type (WT), delta, mu, and omicron RBD from samples at 3 different timepoints including post-2nd dose (P2) 1-3 months, pre-booster which is P2 5 months onwards and post-booster 1 month, with plasma-based cpVNT. Each dot represents the average % blocking of one sample. The medians are indicated as black lines. The table represents number of samples tested from each timepoint for the respective vaccines. Kruskal-Wallis tests with Dunn's multiple comparisons tests were performed between different timepoints, where **P* < 0.05, ***P* < 0.01, ****P* < 0.001, *****P* < 0.0001.

S4 Figure. Comparisons of nAb responses against WT/delta and omicron of individual samples.

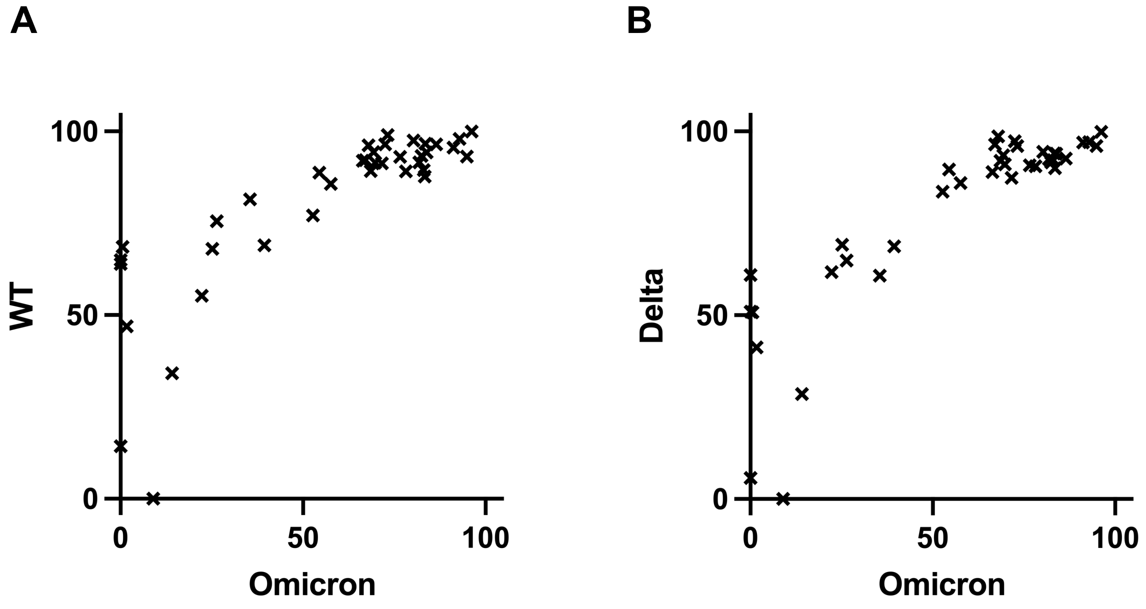

The nAb response data of the 38 samples from various vaccination timepoints that were tested against both WT, delta, and omicron variants were extracted and cross-compared. (A) The individual percent nAb blocking of WT was plotted against that of omicron. (B) The individual percent nAb blocking of delta was plotted against that of omicron.

Reference

[1] Kongsuphol P, Jia H, Cheng HL, Gu Y, Shunmuganathan BD, Chen MW, et al. A rapid simple point-of-care assay for the detection of SARS-CoV-2 neutralizing antibodies. Commun Med 2021;1:1–12. https://doi.org/10.1038/s43856-021-00045-9.

[2] Lim SM, Cheng HL, Jia H, Kongsuphol P, Shanmuganathan BD, Chen MW, et al. Finger stick blood test to assess post vaccination SARS-CoV-2 neutralizing antibody response against variants. Bioeng Transl Med 2022:e10293. https://doi.org/10.1002/BTM2.10293.
